# Phenomenology of Burnout Syndrome and Connection Thereof with Coping Strategies and Defense Mechanisms among University Professors

**DOI:** 10.3390/ejihpe10010008

**Published:** 2019-09-25

**Authors:** Xin Zhao, Shaojie Ding

**Affiliations:** 1 School of Education Science, Zhoukou Normal University, Zhoukou 466000, China; 20171035@zknu.edu.cn; 2 Ukraine-China center, Kharkiv Petro Vasylenko National Technical University of Agriculture, Kharkiv 61000, Ukraine

**Keywords:** burnout syndrome, emotional exhaustion, depersonalization, personal accomplishment, coping strategies, defense mechanisms, university professors

## Abstract

This article is dedicated to the phenomenology of burnout syndrome among university professors. The features of the manifestation of burnout syndrome and its components in university professors are described. The relationship between the burnout components and coping strategies among teachers is considered. The differences in the leading coping strategies among teachers with high and low levels of burnout syndrome are revealed. The relationship between the components of burnout and protective mechanisms among teachers is revealed. The specifics of the manifestation of protective mechanisms among teachers with high and low levels of burnout syndrome is studied. The factor structure of the interconnection of burnout components, coping strategies and protective mechanisms among university professors is presented.

## 1. Introduction

The problem of the influence of one’s occupation on a person, for the purpose of a more detailed study of various aspects of personality deformation, is relevant for contemporary psychological science. The most common form of manifestation of this kind of deformation is emotional burnout syndrome. For the first time, the term “staff burnout syndrome” was suggested by the American psychiatrist Freudenberger (1974) [1] to describe a psychological state of volunteers in the field of psychiatry, which manifests itself in the form of psycho-emotional exhaustion, disappointment and refusal of work.

Burnout syndrome has a varied phenomenology: mental and physical health, emotional exhaustion, professional maladaptation, reduced productivity and professional motivation, formation of negative attitudes towards professional activities, violations of the system of interpersonal relationships, formation of dependent behavior, etc.

The burnout phenomenon is most often observed in representatives of so-called helping professions, namely, health workers, teachers, social workers, psychologists and psychotherapists. However, the list of occupations related to the group of burnout development risk is constantly expanding.

The first scientific publications dedicated to the burnout issue were of a theoretical nature. With the advent of the Staff Burnout Scale for Health Professionals [2] and the publication of the Maslach Burnout Inventory [3], experimental study on burnout syndrome was started.

There are various conceptual approaches to defining the concept of burnout, its structural components and special developmental aspects. Therefore, burnout is considered: as a process of a four-stage progressive disenchantment with the profession as a result of the conflict between the internal idealist expectations of the individual and the everyday reality [4]; as a consequence of the unsuccessful search for the meaning of life in the professional sphere [5]; from the point of view of the “theory of conservation of resources”, whereby “burnout” is developed as a result of a loss or lack of the resources required for the proper functioning of resources [6]; as a consequence of a narcissistic personality disorder, a tendency to excessive idealization of professional activity and constant disappointment in it [7]; as a result of the whole set of false expectations that do not correspond to the real occupational situation [8,9]; and as a manifestation of a non-existential installation in attitude to life [10].

There is a number of burnout syndrome models that describe the stages of its development: a one-factor burnout syndrome model [11], whereby burnout is a state of physical, emotional and cognitive depletion caused by prolonged stay in emotionally overburdened situations; a two-factor burnout model [12], whereby burnout consists of emotional exhaustion and depersonalization; a three-factor model, including emotional exhaustion, depersonalization and reduction of personal achievements [13]; a four-factor model [14,15] wherein one of the elements (emotional exhaustion, depersonalization, or reduction of personal achievements) is divided into two separate factors.

In addition to the aforementioned concepts, there are so-called procedural models of burnout syndrome, which consider this phenomenon as a dynamic process, characterized by an increasing degree of severity of its manifestations [16,17,18].

The consideration of the three-component structure of burnout syndrome [13], which includes emotional exhaustion, depersonalization, and reduction of personal achievements, is traditional. Emotional exhaustion manifests itself in a sensation of emotional over-tension, emptiness, and exhaustion of personal emotional resources. Depersonalization is a tendency to develop a negative, cynical attitude towards subjects of one’s professional occupation and to establish impersonal and formal contacts. The reduction of personal achievements manifests itself in reduction in the sense of competence in work, dissatisfaction with oneself, a sense of diminishing the value of one’s activity, neglecting official duties, and negative self-perception in the professional setting.

Based on this concept, Golembiewski & Munzenrieder (1988) [18] proposed distinguishing between three degrees and eight burnout phases, which differ in the ratio of indicators to all three factors, diagnosed using the Maslach Burnout Inventory (MBI) questionnaire [13]. The proposed model allows the selection of low (phases 1–3), medium (phases 4–5) and high degrees of burnout (phases 6–8). In the last phase, there is a pronounced exhaustion of emotional resources, low self-esteem in personal achievements, and frustration with the occupation.

Burnout syndrome is of an international and intercultural nature [19]. Cross-country studies have shown the global nature of burnout, despite differences in working conditions and cultural traditions.

The World Health Organization has recognized burnout syndrome as an issue requiring medical intervention. In the International Classification of Diseases 11th Revision for Mortality and Morbidity Statistics (ICD-11 MMS, 2018 [20]), burnout syndrome is referred to by separate diagnostic taxon—Problems associated with employment or unemployment and are encrypted QD85: Burn-out. Burn-out is a syndrome conceptualized as resulting from chronic workplace stress that has not been successfully managed. It is characterized by three dimensions: (1) feelings of energy depletion or exhaustion; (2) increased mental distance from one’s job, or feelings of negativism or cynicism related to one’s job; and (3) reduced professional efficacy. Burn-out refers specifically to phenomena in the occupational context and should not be applied to describe experiences in other areas of life [20].

Many studies show that teachers are increasingly experiencing high feelings of stress, which affect the quality of education, as well as the relevance of developing emotional intelligence, which helps prevent these negative feelings from appearing [21,22].

The occupation of teacher or professor requires personal involvement from someone with specialist, developed, empathic and communicative abilities, with a focus on communication and interaction [23]. The multifunctional nature of the professional occupation of a teacher (combining educational, scientific, methodological, organizational, and educational functions), the intensity of work and increased psycho-emotional load make it possible to refer to stress-related occupations with a high level of psycho-emotional tension [24]. Chronic stress, the feeling of unmet work needs, as well as age and personality, are determinants of teachers’ health and mental well-being [22,25].

A study by Puertas-Molero et al. (2018) [26] shows that emotional intelligence and non-verbal communication are two relevant factors in the prevention of burnout syndrome, and as a result can help to ensure the mental wellbeing of university teachers. It has been shown that high emotional competence enhances the ability to cope with symptoms of burnout, by reducing the experience of stress [27,28].

A study by Quraishi et al. (2018) [29] provides an insight into the coping strategies used by university teachers in Pakistan. Research revealed the differences in coping strategies of teachers based on their age group. To cope with stress, positive reinterpretation and growth was most frequently used by teachers, followed by turning to religion, planning, suppression of competing activities, active coping, restraining coping, seeking social support for emotional reasons, acceptance, mental disengagement, alcohol-drug disengagement, focusing on venting of emotions, seeking social support, behavioral disengagement, and denial. Young teachers used two stress coping strategies, namely, positive reinterpretation and growth and turning to religion significantly less than the elder group. Teachers of the middle age group used the stress coping strategy of mental disengagement significantly more than other age groups.

A study by Guerrero Barona (2003) [30] examines the relationship between the levels of burnout and various stress management methods, used by university professors. It is revealed that professors with a high level of emotional exhaustion have predominating passive coping strategies, which include emotional exhaustion, behavioral distancing and acceptance of the situation. Professors with a high level of personal achievement reduction have predominating active methods, including active opposition, planning, searching for instrumental and social assistance, and a positive re-evaluation of the situation.

Pu et al. (2017) [31] investigated the relationship between work–family conflict and job burnout as well as the potential mediation/moderation effects of psychological capital. According to the results, work–family conflict and psychological capital were both significantly correlated with job burnout.

However, the existing works on the issue of professional burnout among professors in higher educational institutions are scarce. Due to this fact, the further deepening and expansion of this problem, in particular, the study of the features of the manifestation of burnout syndrome among university professors seems to be relevant for the purpose of further development of psychological recommendations aimed at preserving professional health and preventing the formation of burnout syndrome in this category of professionals.

## 2. Materials and Methods

### 2.1. Participants

Two hundred and four professors from Xinyang Normal University, Henan Normal University and Zhengzhou University, wherein there were 92 men (45.1%) and 112 women (54.9%), took part in the study.

Respondents’ age range: from 25 to 35 years old, 63 persons (30.88% ± 3.23%); 36–45 years old, 95 persons (46.57% ± 3.49%), 46–55 years, 46 persons (22.55% ± 2.93%).

Experience of work at the University: less than 5 years, 46 persons (22.55% ± 2.93%); 5–15 years, 97 persons (47.55% ± 3.5%); 16–25 years, 52 persons (25.49% ± 3.05%); more than 26 years, 9 persons (4.41% ± 1.44%).

Respondents occupied the following positions at the university: associate professor, 126 (61.76% ± 3.40%); full professor, 78 persons (38.24% ± 3.40%).

Distribution of respondents according to majors: humanities, 61 persons (29.9% ± 3.21%); social sciences, 52 persons (25.49% ± 3.05%); engineering sciences, 49 persons (24.02% ± 2.99%); natural sciences, 42 persons (20.59% ± 2.83%).

### 2.2. Procedure

The study procedure included a preliminary discussion, psycho-diagnostic study, processing, analysis and interpretation of the received data. The preliminary discussion was aimed at introducing professors to the purpose and procedure of the study. All subjects gave their informed consent for inclusion before they participated in the study. Participation in the study was voluntary and was executed by adhering to the ethical principles of conducting the psychological study. The study was conducted in accordance with the Declaration of Helsinki, and the protocol was approved by the Biomedical Ethics Committee of Zhoukou Normal University (Ethical review No. 2019010).

### 2.3. Data Analysis

Correlation analysis (*r*-coefficient of Pearson correlation) was used to reveal the relationship between the structural components of burnout syndrome and coping strategies, as well as the mechanisms of psychological protection among respondents. To determine the differences in the frequency of the burnout components depending on the gender of the professors, the *φ*-criterion for Fisher’s angular transformation was used. To determine the differences in the features of coping behavior and the mechanisms of psychological protection among respondents with high and low burnout levels, Student’s *t*-criterion for independent samples was used.

For the purpose of determining the totality of internal interconnections and possible causal relationships in the empirical material obtained, a factor analysis was carried out. In this case, factor analysis using the method of the main components (Varimax rotation, Kaiser Normalization) was used, enabling the creation of a compact content description of the phenomenon under study, based on the formalization of large information arrays.

All data were processed anonymously using a series of codes and a 95% confidence level for all results (*p* < 0.05). The analysis was performed by using the statistical computer program IBM SPSS Statistics v. 22.0 (SPSS Inc., Chicago, IL, USA).

### 2.4. Instruments

The following tools were used at the psycho-diagnostic study stage: Maslach Burnout Inventory—Educators Survey (MBI-ES) [13], The Coping Inventory for Stressful Situations (CISS) [32], and The Life Style Index [33].

The Maslach Burnout Inventory (MBI) [13] is aimed at identifying emotional burnout personality syndrome. The MBI-ES is a version of the original MBI for use with educators, including teachers, administrators, other staff members, and volunteers working in any educational setting. The questionnaire consists of 22 statements about feelings and experiences related to work. The respondents were asked to assess the proposed statements according to the following scale: 0—never; 1—very rarely; 2—rarely; 3—sometimes; 4—often; 5—very often; 6—always. According to the key, the scores were calculated on three scales: emotional exhaustion, depersonalization and reduced personal accomplishment.

The Coping Inventory for Stressful Situations (CISS) [32] is a 48-item instrument used to measure three basic coping strategies with 16 items per scale: Task-Oriented, Emotion-Oriented, and Avoidance. The Avoidance Scale contains two subscales: Distraction and Social Distraction. Items are scored on a five-point Likert scale: from 1 (not at all) to 5 (very much). Scores for all items per scale are summed to form scale scores; higher scores indicate a greater use of that particular coping strategy.

The Life Style Index [33] is a 97-item questionnaire with statements to which the test-taker responds “usually true” or “usually not true”. From these responses, scores for eight defense mechanisms are derived, including compensation, denial, displacement, intellectualization, projection, reaction formation, regression, and repression. Each of the aforementioned protective mechanisms corresponds to 10–14 statements describing the personality reactions of a person arising in different situations. According to the key, the number of positive responses for each of the eight scales is calculated. The received raw points are transferred to the walls, on the basis of which the profile of the ego-defense is made and the degree of their tension is evaluated.

## 3. Results

Regarding the results of the study, according to the MBI-ES questionnaire [13] and the dynamic phase burnout model, proposed by Golembiewski & Munzenrieder (1988) [18], the following data were received. The high level of burnout syndrome, corresponding to phases 6–8, was diagnosed in 65 professors (31.9% ± 3.26%); the average burnout rate, corresponding to phases 4–5, was detected in 83 professors (40.7% ± 3.44%) and the low level, corresponding to phases 1–3 of burnout, was diagnosed in 56 respondents (27.4% ± 3.12%).

The high level of emotional exhaustion, characterized by a feeling of high psycho-emotional stress and a deficit of emotional resources, was diagnosed in 37.8% ± 3.39% of respondents. At the same time, a high level of emotional exhaustion was found in 30.4% ± 4.8% of men and 43.7% ± 4.69% of women (calculation from the total number of men and women in the sample, respectively). It is hereby found that among female professors this component of burnout is more commonly found than among male professors (*φ* = 1.967, *p* < 0.05).

The high level of severity of the “depersonalization” component, involving tendencies to develop a negative, cynical attitude towards subjects of one’s professional activity and to establish impersonal and formal contacts, was revealed in 29.4% ± 3.19% of professors. The development of depersonalization in professors is related to a high emotional and communicative load and a wide network of contacts of different levels. At the same time, a high level of depersonalization was found in 36.9% ± 5.03% of men and 23.2% ± 3.99% of women (calculation from the total number of men and women in the sample, respectively). It is hereby found that male professors are more inclined to develop a high level of depersonalization than their female colleagues (*φ* = 2.142, *p* < 0.05).

Reduced personal accomplishment, characterized by a decrease in the sense of one’s own competence as a specialist, a feeling of hopelessness in their professional future, neglect of official duties, and a negative self-perception in a professional setting, was found at a high level in 31.9% ± 3.26% of professors.

To identify the relationship between the structural components of burnout and coping strategies, used by university professors, a correlation analysis was performed, the results of which are given in Table 1.

Positive correlations between the components of emotional exhaustion and depersonalization (*r* = 0.681, *p* < 0.01), emotional exhaustion and reduced personal accomplishment (*r* = 0.245, *p* < 0.01), and depersonalization and reduced personal accomplishment (*r* = 0.388, *p* < 0.01) were found. The positive correlations between these elements indicate the significance of each structural component in the formation of burnout syndrome in professors.

The following correlations were found between burnout components and coping strategies: emotional exhaustion correlates positively with emotionally oriented copying (*r* = 0.342, *p* < 0.01) and with distraction (*r* = 0.278, *p* < 0.01) and is negatively correlated with social distraction (*r* = −0.263, *p* < 0.01). The indicators of emotional exhaustion are increased both with the excessive emotional inclusion in the stress situation by the respondents and in case of distraction from the situation by switching to other activities. At the same time, there is a decrease in the severity of emotional exhaustion when using a coping strategy focused on finding a social support.

A positive correlation was found between the depersonalization component and the emotion-oriented coping strategy (*r* = 0.230, *p* < 0.05), and negative correlations were found between depersonalization and the task-oriented coping strategy (*r* = −0.205, *p* < 0.05), as well as between depersonalization and social distraction (*r* = −0.225, *p* < 0.05). The development of negative or formal attitude to subjects of one’s occupation correlates with excessive emotional experience of a stressful situation, whereas active inclusion in the solution of a problem situation and seeking social support are accompanied by a decrease in the depersonalization severity.

The component of reduced personal accomplishment negatively correlates with the task-oriented coping strategy (*r* = −0.275, *p* < 0.05) and social distraction (*r* = −0.182, *p* < 0.05). The received results demonstrate the preservation of professional motivation and confidence in their professional competence when referring to constructive coping strategies aimed at solving problem situations and seeking social support.

Task-oriented coping strategy turned out to be directly related to social distraction (*r* = 0.243, *p* < 0.01). Emotionally oriented coping correlate positively with coping, aimed at avoidance (*r* = 0.301, *p* < 0.01) and its subclass, distraction (*r* = 0.510, *p* < 0.01).

The results of the comparative analysis of mean values for coping strategies among professors with different degrees of emotional burnout are given in Table 2.

According to the received results, higher emotionally-oriented coping (*p* < 0.001) and avoidance (*p* < 0.001) values are revealed in the group of professors with a high-burnout level, compared with their low-burnout colleagues. In the group of professors with a low-burnout level in comparison with the first group, indicators of coping strategies focused on problem solution (*p* = 0.007) and social disturbance (*p* = 0.008) prevail.

The results of the correlation analysis between the structural components of burnout and the mechanisms of psychological protection among professors are given in Table 3.

We turn our attention to a more detailed consideration of the revealed significant correlations between the components of burnout and the ego-protections of the professors. Emotional exhaustion positively correlates with protective mechanisms such as repression (*r* = 0.466, *p* < 0.01), regression (*r* = 0.314, *p* < 0.01) and displacement (*r* = 0.437, *p* < 0.01). This burnout component is also related to a denial (*r* = −0.276, *p* < 0.01), compensation (*r* = −0.182, *p* < 0.05) and reaction formation (*r* = −0.210, *p* < 0.05). Psycho-emotional tension and a feeling of a lack of emotional resources among professors are accompanied by displacement from the consciousness of negative experiences, actualization of less mature behavioral stereotypes, impulsiveness and weakening of emotional and will control, or discharge of negative emotions to safer objects (for instance, the students). The updating of the protective mechanisms aimed at rejecting unacceptable experiences, the formation of opposing negative feelings of aspirations or overcoming frustration situations through self-assertion in other spheres of activity is accompanied by a decrease in psycho-emotional tension and contributes to the preservation of professors’ emotional resources.

Depersonalization correlates positively with ego–protections such as repression (*r* = 0.295, *p* < 0.01), regression (*r* = 0.318, *p* < 0.01) and displacement (*r* = 0.371, *p* < 0.01). A negative correlation was detected between depersonalization and denial (*r* = −0.282, *p* < 0.01). Formalism and negative attitudes toward students and colleagues are accompanied by the use of protective mechanisms aimed at suppressing negative experiences, which can find a release in unmotivated outbursts of irritability, or are accompanied by a decrease in emotional and will control and the discharge of negative emotions to the parties in charge of the activity. Reorientation of attention to other spheres of activity and neglecting negative emotions and frustrating circumstances are accompanied by the preservation of a more positive attitude towards students and colleagues.

Reduced personal accomplishment positively correlates with regression (*r* = 0.274, *p* < 0.01) and displacement (r = 0.303, *p* < 0.01), and is inversely correlated with denial (*r* = −0.297, *p* < 0.01). The reduction of professional motivation and doubts in their own professional competence are related to the treatment of non-constructive and immature forms of overcoming stressful situations, or the spontaneous discharge of negative experiences in situations of safe social interaction. Distortion of the perception of reality by ignoring unacceptable circumstances is accompanied by a decrease in the severity of the reduction of personal achievements.

The results of the comparative analysis of ego-protection tension among professors with different degrees of emotional burnout are given in Table 4.

As it follows from Table 4, professors with a high burnout level have a higher level of intensity of ego-defenses such as displacement, regression, projection and reaction formation, compared with their counterparts without burnout, while professors with a low burnout level have a high level of intensity of denial.

As a result of the factor analysis (main component method, varimax rotation with Kaiser Normalization), wherein the scales of all three methods were included, only one factor including components of burnout syndrome was received, which explains 23.7% of the dispersion. This factor turned out to be bipolar and included the following most significant elements in the general structure: emotional exhaustion (0.789), depersonalization (0.701), repression (0.691), displacement (0.688), general burnout index (0.665), regression (0.636), reduced personal accomplishment (0.496), emotion-oriented coping strategy (0.493), distraction (0.385), social distraction (−0.422), denial (−0.380), and task-oriented coping strategy (−0.316). The manifestation of this factor reflects the higher factor load of the components of emotional exhaustion and depersonalization in the general structure of burnout syndrome; the relationship of the formation of burnout with the high intensity of protective mechanisms such as repression, displacement, and regression; as well as the activation of the emotion-oriented coping strategy and coping strategies of distraction. The activation of coping strategies focused on solving the problem situation and seeking social support, as well as the protective mechanism of denial, is accompanied by a reduction in the severity of the burnout components.

## 4. Discussion

Following the results of the study, 31.9% of the professors showed a high level of burnout syndrome, confirming the expressed emotional exhaustion, development of negative attitudes towards the parties in charge of the activity, low self-esteem in personal achievements, and a decrease in professional motivation and the quality of performance of official duties. The sustained manifestation of the individual components of burnout characterizes 40.7% of the respondents, and only 27.4% of the professors have not been diagnosed with clear signs of burnout syndrome. The received results confirm that more than half of the professors are in a state of psycho-emotional tension, which contributes to the development of increased fatigue, emotional exhaustion and is a risk factor for further somatization of the aforementioned symptoms. These results confirm earlier studies showing an increasing tendency for university teachers to develop burnout syndrome [23,30].

It is hereby found that female professors are more inclined to high emotional inclusion in a communicative process with students than their male colleagues, resulting in a more rapid exhaustion of emotional resources. Male professors are more inclined to establish formal and deformed contacts in the process of their professional activity, which is a way of saving emotional resources. Similar results regarding the gender-specific manifestations of burnout syndrome were obtained in studies by Maslach, Schaufeli and Leiter (2001) [34].

The professors’ tendency to underestimate their professional achievements may be related to high communicative load, the need for continuous improvement of the teaching process and the use of new and more effective teaching methods, and shortage of time for scientific and research work and professional development, which requires additional energy resources. These risk factors for the development of burnout syndrome among university professors were considered in studies on similar topics [29,35,36,37,38].

The formation of burnout syndrome is related to the activation of the individual’s adaptive resources. The most important forms of personal adaptation processes are coping strategies and psychological defense mechanisms.

The interrelation between burnout syndrome components and the leading coping strategies of the professors was revealed. Several studies have found a positive correlation between high levels of burnout and stress passive resistance tactics [24,29,34]. The indicators of emotional exhaustion and depersonalization are increased, when referring to emotionally-oriented coping, suggesting the immediate emotional response to negative experiences. The use of constructive coping strategies by professors, namely the active inclusion of a problem situation and social support into the search for a solution, is accompanied by a decrease in the severity of all components of burnout. Therefore, it can be assumed that professors’ formation of burnout syndrome is related to reference to non-constructive or passive coping strategies that involve excessive emotional inclusion in a problem situation, its emotional experience with a tendency to self-incrimination, and avoidance of active actions with regards to its resolution. Professors with a low level of burnout are more focused on actively solving a problem situation, as well as seeking social support.

The interrelation between burnout syndrome components and the Ego-defenses of professors were revealed. The professors’ emotional exhaustion and the formation of negative attitudes towards students and colleagues are intensified in case of pushing away negative experiences out of consciousness, updating less mature behavioral patterns and spontaneous discharge of negative emotions in situations of safe social interaction. The formation of aspirations opposite to negative feelings or overcoming frustrating situations through self-affirmation in other areas of activity are accompanied by a decrease in psycho-emotional tension and contribute to the preservation of professors’ emotional resources. The distortion of the perception of reality by ignoring unacceptable circumstances is accompanied by a decrease in all components of burnout syndrome. In general, professors with a high level of burnout, compared with their colleagues without burnout, are characterized by a higher overall level of Ego-defense intension and the predominance of protective mechanisms. It can be assumed that the manifestation of burnout syndrome is accompanied by activation of the entire system of psychological defense mechanisms [25,33].

The factor analysis enabled the revelation of the cause-and effect relationship between the burnout components, coping strategies and Ego-defenses of professors. The formation of all three components of burnout syndrome is related to a high intensity of the protective mechanisms of repression, regression and displacement, as well as with reference to passive coping strategies, aimed at emotional response and distraction. In addition, on the contrary, active coping strategies aimed at finding a solution to a problem situation and seeking social support, as well as “distortion” of the perception of a problem situation by denying its negative components, contribute to the reduction of emotional burnout.

Finally, several limitations of the current study should be mentioned. Firstly, the question of a causal relationship between burnout components and defense mechanisms remains open. The question arises as to whether immature defense mechanisms are predictors of burnout or they are activated as a result of the formation of burnout symptoms. Secondly, in this study, the role of gender and age aspects, as well as work experience in choosing coping strategies in professional activities among university professors, is not considered. Thirdly, it is of interest to study the characteristics of the manifestation of burnout syndrome and coping behavior among university professors, depending on their position and major. In particular, in further studies it would be interesting to verify the correctness of the hypothesis that humanitarian and social sciences professors may be more prone to the development of burnout syndrome than their colleagues teaching natural and technical disciplines.

Thus, the above problems and limitations of the current study may be the subject of further scientific developments. Further study of the relationship between the characteristics of coping behavior and burnout syndrome among university professors will help to develop recommendations and a psychocorrectional program aimed at increasing stress resistance, developing skills of constructive coping behavior and enhancing the adaptive potential of university professors.

## 5. Conclusions

The results of this study showed that more than half of professors showed symptoms of burnout syndrome. The identified relationships between the components of burnout, coping behaviors, and Ego-defenses among professors enabled a more complete understanding of burnout phenomenology and the role of the aforementioned adaptation processes in its manifestation among university professors.

Professors’ formation of burnout syndrome is related in reference to non-constructive or passive coping strategies, which involve excessive emotional inclusion in a problem situation, its emotional experience with a tendency to self-incrimination, and avoidance of active actions with regards to its resolution. Professors with a high level of burnout are characterized by a higher level of Ego-defense intension and the predominance of protective mechanisms.

The received results confirm that university professors’ development of skills for constructive coping with stressful situations is an important condition for increasing the individual’s adaptive potential and prevention/reduction of burnout symptoms.

## Figures and Tables

**Table 1:** Correlation matrix of the relationship between the components of burnout and coping strategies used by professors.

	1	2	3	4	5	6	7
1. Emotional exhaustion							
2. Depersonalization	0.681 **						
3. Reduced personal accomplishment	0.245 **	0.388 **					
4. Task-oriented coping strategy	−0.019	−0.205 *	−0.275 *				
5. Emotion-oriented coping strategy	0.342 **	0.230 *	0.083	−0.118			
6. Avoidance	0.029	−0.028	−0.007	0.103	0.301 **		
7. Distraction	0.278 **	0.130	0.083	0.001	0.510 **	0.840 **	
8. Social distraction	−0.263 *	−0.225 *	−0.182 *	0.243 **	−0.163	0.589 **	0.120

Note: * *p* < 0.05; ** *p* < 0.01.

**Table 2:** Comparative analysis of the severity of coping strategies among professors with high and low levels of burnout syndrome.

	Group 1		Group 2		*t*	*p*
	*M*	*SD*	*M*	*SD*		
Task-oriented coping strategy	56.82	8.423	60.65	6.793	2.764	0.007
Emotion-oriented coping strategy	44.11	8.750	32.14	7.632	−8.037	<0.001
Avoidance	44.04	8.655	42.77	7.180	−0.880	0.381
Distraction	20.88	6.322	17.00	4.613	−3.886	<0.001
Social distraction	15.61	3.055	17.06	2.850	2.707	0.008

Note: Group 1: Professors with a high level of burnout. Group 2: Professors with a low level of burnout.

**Table 3:** Correlation Matrix of interconnection between burnout components and mechanisms of professors’ psychological protection.

	1	2	3	4	5	6	7	8	9	10
1										
2	0.681 **									
3	0.245 **	0.388 **								
4	−0.276 **	−0.282 **	−0.297 **							
5	0.466 **	0.295 **	0.145	−0.206 *						
6	0.314 **	0.318 **	0.274 **	0.024	0.359 **					
7	−0.182 *	−0.035	−0.046	0.368 **	0.001	0.248 **				
8	−0.008	0.041	0.145	0.111	0.155	0.278 **	0.306 **			
9	0.437 **	0.371 **	0.303 **	−0.075	0.430 **	0.710 **	0.176	0.308 **		
10	−0.002	0.013	−0.037	0.239 **	0.047	0.081	0.165	0.123	−0.036	
11	−0.210 *	−0.106	−0.115	0.219 *	−0.038	−0.033	0.390 **	0.400 **	0.011	0.002

Note: * *p* < 0.05; ** *p* < 0.01; 1: emotional exhaustion; 2: depersonalization; 3: reduced personal accomplishment; 4: denial; 5: repression; 6: regression; 7: compensation; 8: projection; 9: displacement, 10: intellectualization; 11: reaction formation.

**Table 4:** Comparative analysis of the severity of mechanisms of psychological protection among professors with high and low levels of burnout syndrome.

	Group 1		Group 2		*t*	*p*
	*M*	*SD*	*M*	*SD*		
Denial	74.27	19.052	83.06	18.083	2.602	0.010
Repression	71.11	20.274	37.43	31.771	−6.823	<0.001
Regression	52.00	29.175	36.60	27.993	−2.959	0.004
Compensation	64.29	32.138	56.89	29.736	−1.314	0.191
Projection	58.25	25.395	36.15	26.254	−4.686	<0.001
Displacement	54.68	30.904	32.71	23.297	−4.450	<0.001
Intellectualization	56.50	27.076	62.32	26.100	1.203	0.231
Reaction formation	72.09	28.916	54.94	30.973	−3.131	0.002

Note: Group 1: Professors with a high level of burnout. Group 2: Professors with a low level of burnout.
